# *In situ* response of Antarctic under-ice primary producers to experimentally altered pH

**DOI:** 10.1038/s41598-019-42329-0

**Published:** 2019-04-15

**Authors:** Vonda J. Cummings, Neill G. Barr, Rod G. Budd, Peter M. Marriott, Karl A. Safi, Andrew M. Lohrer

**Affiliations:** 1 National institute of Water and Atmospheric Research, Wellington, New Zealand; 2 National institute of Water and Atmospheric Research, Hamilton, New Zealand

## Abstract

Elevated atmospheric CO_2_ concentrations are contributing to ocean acidification (reduced seawater pH and carbonate concentrations), with potentially major ramifications for marine ecosystems and their functioning. Using a novel in situ experiment we examined impacts of reduced seawater pH on Antarctic sea ice-associated microalgal communities, key primary producers and contributors to food webs. pH levels projected for the following decades-to-end of century (7.86, 7.75, 7.61), and ambient levels (7.99), were maintained for 15 d in under-ice incubation chambers. Light, temperature and dissolved oxygen within the chambers were logged to track diurnal variation, with pH, O_2_, salinity and nutrients assessed daily. Uptake of CO_2_ occurred in all treatments, with pH levels significantly elevated in the two extreme treatments. At the lowest pH, despite the utilisation of CO_2_ by the productive microalgae, pH did not return to ambient levels and carbonate saturation states remained low; a potential concern for organisms utilising this under-ice habitat. However, microalgal community biomass and composition were not significantly affected and only modest productivity increases were noted, suggesting subtle or slightly positive effects on under-ice algae. This in situ information enables assessment of the influence of future ocean acidification on under-ice community characteristics in a key coastal Antarctic habitat.

## Introduction

Physical and biogeochemical changes in the world’s oceans associated with anthropogenic greenhouse gas emissions have potential to impact marine organisms and ecosystems^1,2^. Ocean acidification, the decline in seawater pH (and concomitant decline in carbonate saturation state) as the oceans absorb more CO_2_, is anticipated to affect organism function^3^ and alter marine food web dynamics (e.g.^4^). Oceanic pH is predicted to decline by −0.33 pH units by 2090–2099 (relative to 1990–1999 levels) under the current trajectory of the “business as usual” Representative Concentration Pathway emissions scenario (RCP8.5)^5^. This represents a considerably faster rate of change, and lower pH, than at any time in the last 25 million years^6^, raising questions of how organisms, populations and communities will respond to this potential challenge that, in some cases, may transcend adaptation capacity time scales.

The threat of ocean acidification is particularly great in cold water environments, where CO_2_ is absorbed more readily and calcium carbonate minerals are more soluble^7,8^. Absorption of CO_2_ is occurring more quickly in the Southern Ocean than in subtropical oceans, and its water chemistry is changing at a higher rate than previously predicted^9^. That such high latitude regions will experience early ocean acidification, altering benthic and pelagic ecosystems, is a high confidence statement in the most recent Intergovernmental Panel on Climate Change report^10^. Seasonally undersaturated carbonate conditions, predicted for the Southern Ocean in the coming decades (i.e. by 2030 in winter months in the Ross Sea^11^; and by austral summer of 2026–2030 in the Ross Sea, Amundsen Sea and coastal Amundsen Sea^12^), will also spread rapidly in aerial extent and temporal duration - particularly from 2040 onwards when atmospheric CO_2_ is around 450–500 μatm^9^.

Antarctic sea ice supports a diverse community of primary producers and consumers, and represents an important multi-trophic module within the broader marine ecosystem^13^. Sea ice-associated microalgal communities contribute significantly to seasonal production^13^, with estimates of 10–50% of the annual production of polar seas^14^ and as much as 55–65% in ice covered coastal ecosystems^15^. Under-ice algal assemblages are an important food resource, not only to organisms utilising the under-side of the ice, but also to the benthos below, as ice algae and detritus sink down to the seafloor, seeding microphytobenthic communities and providing a major food component for benthic primary consumers^16–18^. In consuming this material, the benthos regenerate nutrients to the water column which, in turn, become available for use by the sea ice communities above (e.g.^19^). Consequently, impacts on such primary producers could have considerable ramifications, not least due to their role in carbon cycling.

In the Ross Sea, coastal sea ice algal communities are dominated by diatoms. Studies of open ocean phytoplankton have noted changes to diatom communities under ocean acidification conditions projected for the end of this century^20^. These include selection for larger species (e.g.^21,22^) and, in Southern Ocean waters, alterations in community size structure and nutrient cycling^23^, and increased growth rates^24^ particularly of larger diatom species^25^. Investigations of the response of sea ice associated communities or species to elevated pCO_2_ concentrations are, however, rare^26^. McMinn^26^ identified three published studies that used temperatures realistic for a sea ice environment (≤0 °C)^27–29^, and concluded that the general response across studies was either a neutral or positive effect on photosynthesis and/or growth. Additionally, a study of single diatom species (*Nitzschia lecointei*) in the laboratory showed reduced fatty acid content (indicative of lower food quality) at −1.8 °C and at 960 μatm pCO_2_ relative to the ambient pCO_2_ treatment (390 μatm)^28^. Experiments on surface dinoflagellate dominated microalgal brine communities within the sea ice *in situ* found a positive effect at pH below 7.5, on growth^27^ and photosynthesis^30^.

Given the prevalence of diatom-dominated ice algae communities in the coastal Ross Sea, their exceedingly high concentrations in spring/early summer (up to 1000 μg L^−1^;^31^), and the fact that algal photosynthesis is a major contributor to pH variation and carbonate saturation state (e.g.^12,32,33^), we expect these communities to play a significant role in carbon uptake and, potentially, in seasonal mitigation of ocean acidification conditions in a high CO_2_ world. Understanding how ocean acidification might affect such processes, and their potential to influence food and nutrient availability in nearby benthic and pelagic ecosystems, was the impetus behind this *in situ* experimental study.

We describe the results of a pH manipulation experiment conducted at Granite Harbour (Ross Sea), that enclosed relatively large patches (0.36 m^2^) of natural sea ice-associated (sympagic) microbial community in chambers deployed to the underside of the sea ice^34^. Seawater was introduced to the chambers at ambient pH levels (7.99), and a range of pH levels expected over the following decades-to end of century (7.86, 7.75, 7.61), equivalent to average pCO_2_ concentrations of 457, 642, 802, and 1166 μatm respectively (Table 1). Fluxes of oxygen and nutrients, along with changes in pH, were assessed daily throughout the experimental period (15 d). At the end of the experiment, comparisons of characteristics of the community associated with the bottom and platelet ice were made between treatments. Continuous measurements of photosynthetically available radiation (PAR) and temperature inside each chamber were taken into account when analysing and interpreting the results. Specifically, we asked how exposure to future projected levels of seawater pH modified sea ice community characteristics and net community primary productivity. Consideration of these effects is key to better understanding consequences of ocean acidification on the functioning of sea ice-associated communities, the potential downstream impacts upon other components of coastal ecosystems, and the mediation of seawater CO_2_ concentrations by seasonal biological uptake and fixation.

**Table 1:** Seawater conditions over the experiment (averages ± SE).

Treatment	Inflow					Outflow				
	pH_T_	pCO_2_	DIC	Ω_Ar_	Ω_Ca_	pH_T_	pCO_2_	DIC	Ω_Ar_	Ω_Ca_
Ambient	7.99 ± 0.002	457.3 ± 7.37	2259.3 ± 2.62	1.2 ± 0.02	1.8 ± 0.03	8.08 ± 0.002	374.9 ± 18.01	2232.4 ± 8.06	1.4 ± 0.05	2.2 ± 0.08
pH low 1	7.86 ± 0.006	641.5 ± 16.28	2301.3 ± 4.48	0.9 ± 0.02	1.4 ± 0.03	8.00 ± 0.005	449.6 ± 25.98	2256.7 ± 8.96	1.2 ± 0.05	1.9 ± 0.09
pH low 2	7.75 ± 0.008	802.3 ± 20.02	2328.0 ± 3.49	0.7 ± 0.02	1.1 ± 0.03	7.96 ± 0.011	504.9 ± 62.24	2269.8 ± 16.73	1.1 ± 0.11	1.7 ± 0.17
pH low 3	7.61 ± 0.006	1166.2 ± 57.47	2373.2 ± 6.97	0.5 ± 0.02	0.8 ± 0.04	7.87 ± 0.012	639.9 ± 90.00	2298.6 ± 18.38	0.9 ± 0.10	1.4 ± 0.16

Inflow = water delivered to the chambers; Outflow = water resident in the chambers for approximately 12 h. Measured pH_T_ is presented at average *in situ* temperature (−1.85 °C), and is an average over the 14 days of the experiment (N = 14). pCO_2_ (μatm), dissolved inorganic carbon (DIC; μmol kg^−1^) and saturation states of aragonite and calcite (Ω_Ar_ and Ω_Ca_) were calculated using measured pH_T_, A_T_, temperature and salinity, and Mehrbach equilibrium constants refit by Dickson and Millero (1987). These calculations were done separately for Days 1, 7 and 14, and the averages ( ± SE) of these three days are presented here. Measured A_T_** = **2348.9 ± 1.86, 2344.5 ± 0.636, and 2342.8 ± 6.4, on Days 1, 7 and 14, respectively (N = 14 chambers/day).

## Results

### General environmental conditions

**Figure 1:**
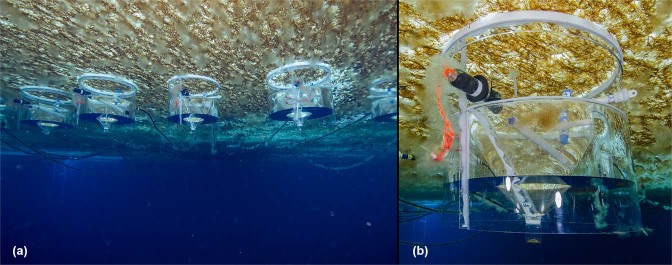
View of chambers deployed *in situ* under the sea ice. (**a**) multiple chambers, umbilical cables linking the chambers with the control system can be seen emerging from the ice hole in the background; (**b**) a close up view of a single chamber. (Photographs: P. Marriott).

**Figure 2:**
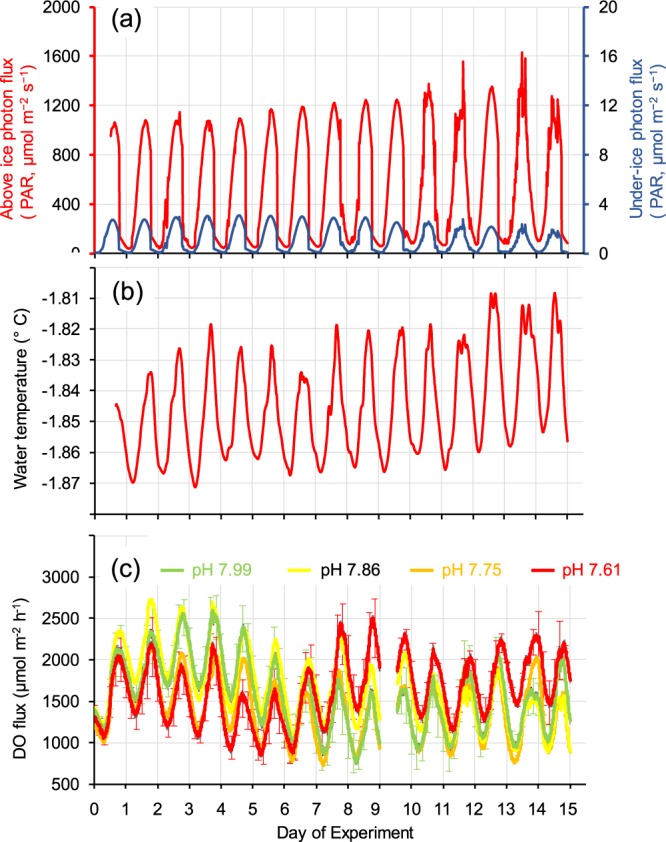
Light, temperature and productivity within the under-ice chambers. Light levels (**a**), sea water temperatures (**b**), and net photosynthetic ice-algal productivity estimates (DO fluxes; (**c**)), at Granite Harbour, 3–18 November 2014. (**a**) Photosynthetically active radiation (PAR) above (red line, left-hand axis) and below (blue line, right hand axis; average of 16 under-ice in-chamber PAR sensors) sea ice; (**b**) average of 16 in-chamber temperature loggers; (**c**) ice-algal productivity estimated from DO loggers present in each chamber (see Methods). All plots are based on 10-minutely data. Error bars on (**c**) are mean per treatment ± 1 SE, and are only given every four hours for the highest and lowest treatments (pH 7.99 and 7.61, respectively) for clarity.

The sea ice at Granite Harbour in November 2014 was 2 m thick, and its under-surface was covered by a dense, diatom-dominated microalgal community (Fig. 1). Light levels below the ice were considerably darker than those above the ice, with levels of under-ice PAR in the chambers generally >2 orders of magnitude lower (Fig. 2a). Over the experimental period, above-ice light levels slowly increased: daily maximum and minimum values and cumulative 24 h light totals all showed significant positive trends (p ≤ 0.0064, r^2^ = 0.61–0.79). However, the under-ice light availability pattern did not match the above-ice pattern, with under-ice light levels showing a modest increase during the first 6 to 7 days and a slow decline over the next 7–8 days (Fig. 2a). At the beginning of the experimental period (Days 0–2), the site was shaded between ~18:30 and ~04:20 h when the sun passed behind adjacent cliffs. By the end of the experiment (Days 14–15), the period of shading was noticeably shorter, from ~19:10 to ~04:00 h, due to the seasonal procession of daily sun arcs. The seawater temperature recorded by the loggers inside the chambers ranged from a low of −1.87 °C to a high of −1.81 °C, and increased very slightly over the 15 days (Fig. 2b). Both PAR and temperature showed pronounced 24 h periodicities, with highs every afternoon (Fig. 2a,b).

### Experimental conditions

**Figure 3:**
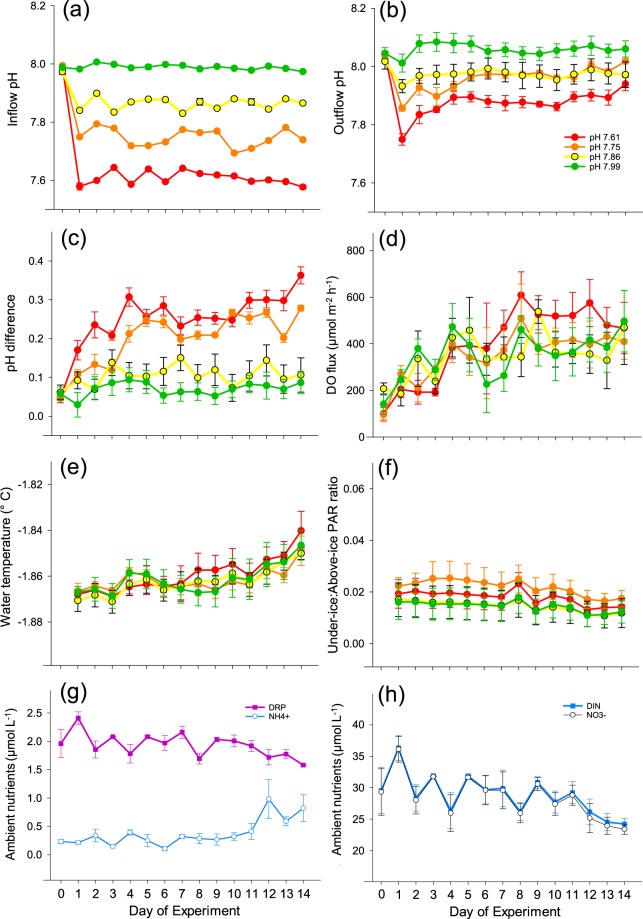
Time series of seawater parameters in each of the four pH treatments during the 15 d experiment. Treatment means ( ± 1 SE) are given in all cases. Panels (**a**,**b**) refer to inflow and outflow pH, respectively. Panels (**c**) and (d) are indicative of CO_2_ uptake and DO production by the enclosed under-ice algal communities, respectively. Panels (**e**–**g**) show trends in environmental variables: chamber seawater temperature (**e**); ratio of light levels, PAR, above and below the sea ice (**f**); concentrations of inorganic nutrients in ambient seawater at the site (**g**,**h**). DRP = dissolved reactive phosphorus; NH_4_^+^ = ammonium nitrogen; NO_3_^−^ = nitrate + nitrite nitrogen; DIN = dissolved inorganic nitrogen.

Prior to any experimental manipulation of pH (pH_T_; total hydrogen scale), the pH of the ambient seawater delivered to the sixteen chambers (hereafter ‘inflow’) was 7.99 ± 0.005 (average ± SE of four header tanks). The three experimentally altered pH treatments initiated on Day 0 (i.e., 7.86, 7.75, 7.61) generated significant differences in inflow seawater pH within 24 h (all treatments significantly different from each other; see Table 1), and these pH differences were able to be maintained for the duration of the experiment (15 d; Fig. 3a).

As air bubbles from the divers entered two of the sixteen chambers during deployment, potentially affecting their under-ice algal communities, these chambers were excluded from our analyses. Consequently, the ambient and pH 7.86 treatments had three replicate chambers, while the remaining treatments (7.75 and 7.61) had four.

### Chamber fluxes

Water samples collected daily from the inflow and outflow of each chamber enabled quantification of pH, salinity, and concentrations of dissolved oxygen (DO) and inorganic nutrients (dissolved inorganic nitrogen, DIN; ammonium nitrogen, NH_4_^+^; nitrate + nitrite nitrogen, NO_3_^−^; reactive phosphorus, DRP; reactive silica, Si(OH)_4_). In all treatment types and throughout the entire 15 d experimental period, change in pH (ΔpH) was positive (i.e., outflow pH was greater than inflow pH; Fig. 3a–c; Table 1). A pronounced increase in ΔpH was observed over time in the two lowest pH treatments (7.61 and pH 7.75). Multiple regression results revealed a combination of four variables—inflow pH, day of experiment, under-ice PAR, and N:P ratio—to be the best predictors of ΔpH (final model p < 0.0001, r^2^_adj_ = 0.8248). Inflow pH (i.e. the experimentally manipulated factor) had the strongest effect of any of the explanatory variables on ΔpH (standardised coefficient of −0.5086), with the negative sign of the coefficient indicating the inverse relationship between inflow pH and ΔpH.

DO flux was positive in all treatments throughout the experiment, indicating net photosynthetic oxygen production by the under ice algal community. Inflow pH and day of experiment were both significant predictors of DO flux (pH inflow p = 0.0175, time p < 0.0001; interaction term not significant, p = 0.4069). Nevertheless, only 18% of the total variation in DO flux was explained by these two variables together. Multiple regression results showed that DO flux was best explained by a combination of five variables: seawater temperature, ratio of under:above-ice PAR, day of experiment, NH_4_^+^ concentration, and N:P ratio (p < 0.0001, r^2^_adj_ = 0.5024; all explanatory variables significant at p < 0.05). Note that inflow pH was not retained in the final model. When included with the other five variables, the standardised coefficient for inflow pH was negative (−0.0252), indicating that reduced pH was linked to higher DO flux, although weakly as the inclusion of pH did not increase the amount of variation explained. The effects of seawater temperature and light ratio on DO flux were much stronger than those of pH, with positive standardised coefficients of 0.4879 and 0.2156, respectively. Increases in ambient NH_4_^+^ concentrations were significantly negatively related to DO flux (−0.1630).

Dataloggers inside the chambers provided further insights into the effects of sunlight intensity on chamber water temperatures and net photosynthetic oxygen production. The productivity of the under-ice community (tracked by DO loggers) exhibited pronounced 24 h cycles, with prominent peaks every afternoon (Fig. 2c). The effect of pH manipulation on net oxygen production was much smaller than the effect of natural daily variation in sunlight intensity, although pH manipulation appeared to gradually increase the baseline productivity rate (Fig. 2c), an observation that was confirmed by our once-daily sampling of the chambers (Fig. 3).

### Sea ice algae-matrix characteristics

**Figure 4:**
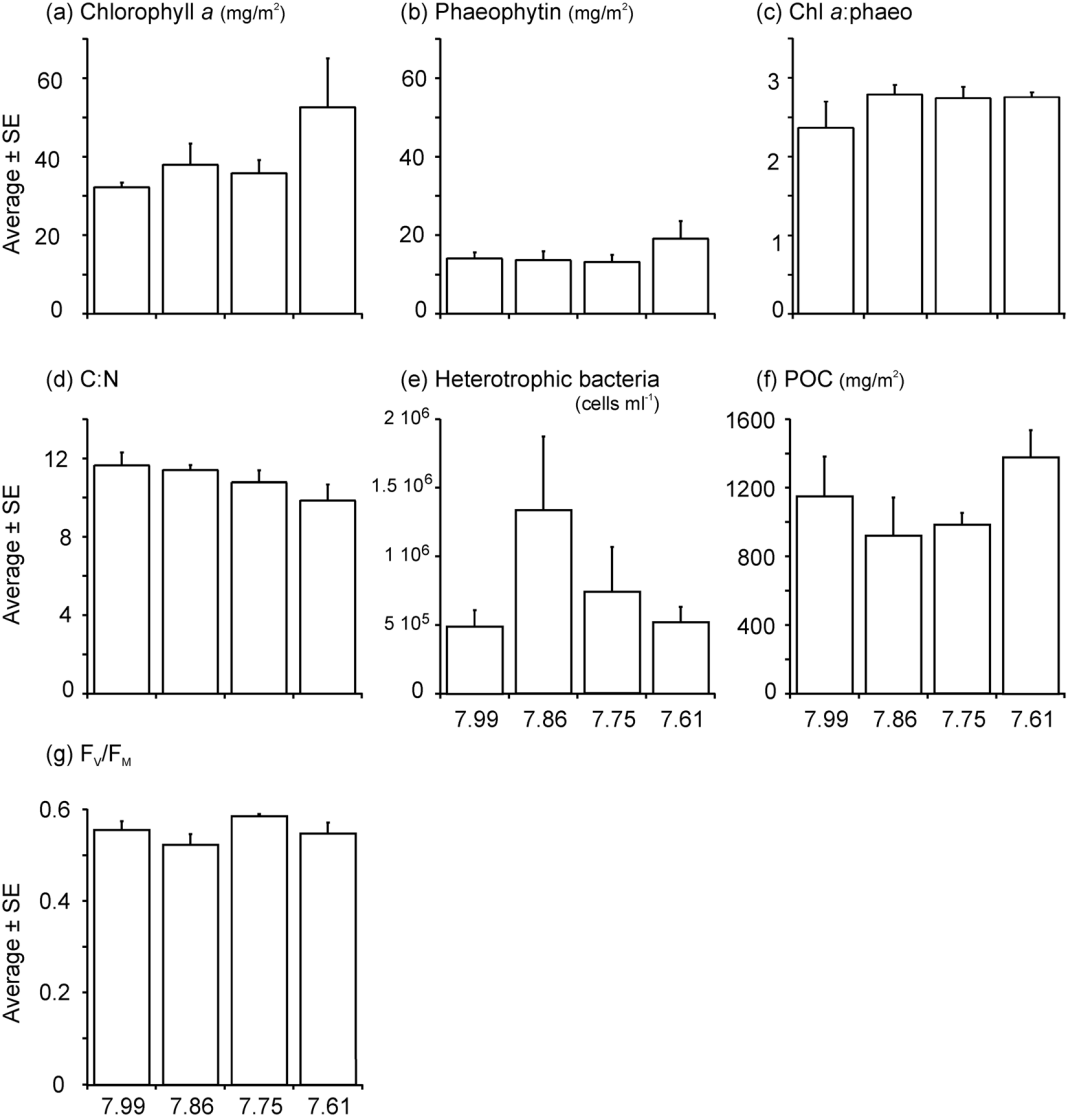
Characteristics of the sea ice matrix associated with the microalgal assemblage at the end of the experiment. POC = particulate organic carbon.

The characteristics of the sea ice matrix associated with the under-ice microalgal assemblage within each chamber were examined at the end of the experiment, from a scrape collected across the central diameter of each chamber (10 cm wide by 70 cm long). Chlorophyll *a* (Chl *a*) and phaeophytin (Phaeo) concentrations were highest and lowest, respectively, and also most variable, in the 7.61 pH treatment (Fig. 4a,b). The ratio of Chl *a*:Phaeo, indicating the relative composition of healthy vs degrading microalgae, was lowest (and most variable) in the ambient treatment chambers, and very similar between the three reduced pH treatments (Fig. 4c). However, there was no significant difference between treatments for Chl *a*, Phaeo, or Chl *a*:Phaeo (Table 2). The very small increase in seawater temperature observed during the 15 d experiment (0.04–0.06 °C) was unlikely to have affected algal biomass.

**Table 2:** Results of PERMANOVA analyses to assess the effect of pH on microbial assemblage characteristics at the end of experiment. None of the variables showed significant differences.

Variable	Variable units	SS	MS	PERMANOVA pseudo-F	P (perm)
Chlorophyll *a*	mg m^−2^	906.68	302.23	1.39	0.25
Phaeophytin	mg m^−2^	87.53	29.18	0.91	0.49
Chlorophyll *a*: Phaeophytin	ratio	0.37	0.12	1.18	0.35
Heterotrophic bacteria	cells mL^−1^	1.45 E^+12^	4.82 E^+11^	1.48	0.23
Particulate organic carbon (POC)	mg m^−2^	8.45 E^+07^	2.82 E^+07^	1.57	0.27
Carbon: Nitrogen	ratio	6.91	2.30	1.43	0.31
F_v_/F_m_	ratio	0.007	0.002	1.90	0.19

There was an indication of a decline in C:N with lowering of pH (Fig. 4d) and heterotrophic bacteria were more abundant in the intermediate pH treatments (Fig. 4e), but their numbers were considerably variable – a feature of most of these measures (particularly POC; Fig. 4f). None of the community characteristics measured at the end of the experiment showed statistically significant differences between treatments (PERMANOVA p > 0.05; Table 2).

**Figure 5:**
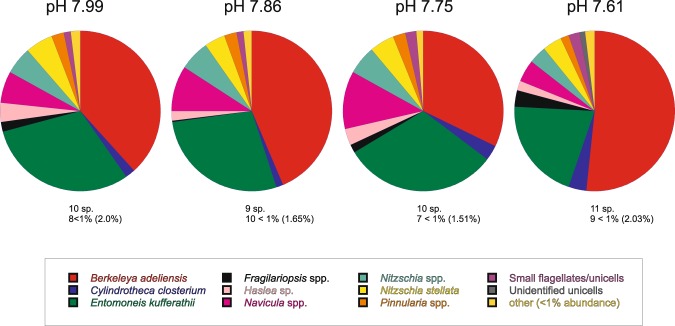
Average percent abundance of the microalgal taxa groups found in each of the pH treatments at the end of the experiment, determined from under ice scrapes. The total number of species found in abundance of >1% (depicted on the pie chart) is provided underneath each pie, as are the number of species found in abundances low than 1% (and the total percentage abundance they collectively contribute).

**Figure 6:**
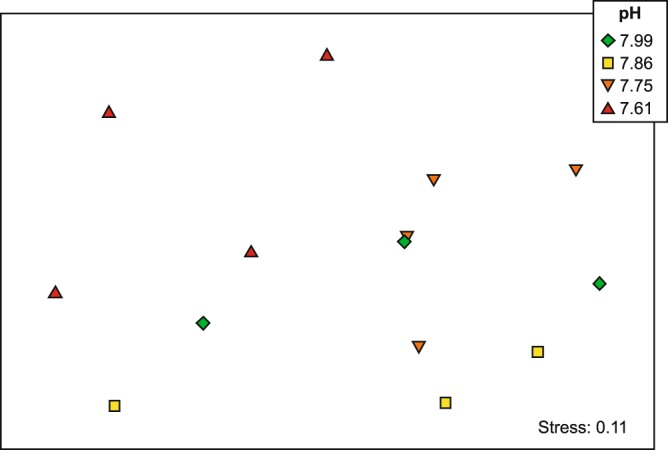
MDS ordination plot of the sea ice microalgae assemblage composition in each pH treatment and chamber. Data are square root transformed. Symbols denote individual chambers; N = 4 chambers for pH 7.75 and 7.61 treatments, N = 3 chambers for pH ambient (7.99) and 7.86 treatments.

Pulse Amplitude Modulated (PAM) fluorometry measurements showed healthy microalgal activity. Mean F_v_/F_m_ values ranged from 0.523 to 0.585 across the pH treatments, with no clear trends apparent (Fig. 4g; Table 2). The under ice microalgal assemblages were comprised of a maximum of 18 different taxa groups, ranging from an average of 16 ± 0.6 in the ambient pH treatment, to 13.5 ± 0.6 in the 7.75 pH treatment. Across all treatments, the community was dominated by the tube forming sympagic diatom species *Berkeleya adeliensis* (Medlin), with the unicellular diatom *Entomoneis kufferathii* Manguin the second most abundant taxa (Fig. 5). On average *B. adeliensis* was most prevalent in the lowest pH treatment (51.6% ± 5.19), while *E. kufferathii* was least prevalent in this same treatment (20.5% ± 1.64). There was, however, no clear (linear) progression of abundances of these taxa from the lowest to the highest pH treatment (Fig. 5). All treatments also contained significant abundances of *Navicula* spp., *Nitzchia* spp, and *Navicula stellata* (Fig. 5; Supplementary Table 1). Only two other taxa groups contributed to the top 90% in SIMPER analysis (PRIMER 7^35^). These were *Haslea* sp. in the ambient and 7.75 pH treatment chambers (3.85 and 3.17% average abundance, respectfully), and *Cylindrotheca closterium* in the 7.61 pH treatment (3.76% average abundance) (Supplementary Table 1). The variability in assemblage composition within and among treatments is also illustrated by the square root transformed MDS, which down weights the importance of the highly abundant species (Fig. 6). The MDS indicated separation of the pH 7.61 treatment chambers in ordination space (Fig. 6). The community from this lowest pH treatment was significantly distinct from the 7.75 treatment chambers (PERMANOVA, p = 0.021), but differed from the 7.86 and 7.99 treatments only at the p < 0.15 significance level (i.e. p = 0.144 and 0.142, respectively).

## Discussion

In this study we enclosed replicate patches of under-ice algal habitat for 15 days, and manipulated seawater pH within each enclosure to levels anticipated to occur in the Southern Ocean in the coming decades. This enabled an *in situ* evaluation of the influence of reduced pH levels (additional pCO_2_) on under ice microalgal photosynthetic productivity and community composition. Measurements of PAR, temperature and ambient nutrient concentrations, made at daily (or greater) temporal frequencies, allowed us to elucidate the potential drivers of photosynthetic DO production and CO_2_ uptake by the under-ice microalgal community, under conditions with and without pH manipulation. The results of our experimental manipulations suggested that the addition of pCO_2_ to this environment stimulated microalgal community photosynthesis (DO production was elevated with reduced pH, and pH changed in a manner suggestive of CO_2_ uptake) yet there was little significant influence on the characteristics of the under ice-associated community.

Seawater temperatures during the experiment at Granite Harbour fluctuated very slightly (0.04–0.06 °C) around a mean value of −1.84 °C. Nevertheless, there was a distinctive 24 h periodicity to these temperature fluctuations as well as a very slight increase over the 15 day experiment (Fig. 2b). Sunlight intensity data from PAR sensors deployed above and below the 2 m thick sea ice layer also exhibited a marked daily periodicity (Fig. 2a). This light cycle was influenced by the height of the sun (which affected the timing of shading from nearby cliffs), and local cloud conditions, with more variability noted on cloudy days. As sea ice thickness did not change appreciably during the two-week period, the slight decrease in under-ice light availability during the latter half of the experiment may have been caused by an increase in under-ice algal biomass and a concomitant increase in microalgae-mediated light absorption. Concentrations of nitrate + nitrite N, total DIN, and DRP decreased during the 15 day experiment (Fig. 2g,h), which is consistent with increasing under-ice algal biomass and a related increase in the demand for nutrients supporting under ice algal productivity. However, the observed pattern in under ice light availability may have also been driven by the accumulation of under-ice algae detritus on the up-facing PAR sensors (less light reached the PAR sensors in chambers containing microalgal communities with a higher proportion of degraded photopigment; correlation between underwater PAR and Chl *a*:Phaeo of *R* = 0.57). Ammonium concentration in ambient seawater samples increased towards the end of the experiment, which was another possible indication of higher levels of detrital under-ice algal material at the site, as NH_4_^+^ is a product of organic matter remineralisation.

During our experiment, the daily average ambient pH conditions recorded at our seawater intake point ~4 m below the sea ice was 7.99 ± 0.002, with average ambient pCO_2_ concentrations of 457 ± 7.37 μatm (Table 1). While these pH values are within the range of those reported close to the seafloor (at 14–20 m) during spring in this region, our calculated pCO_2_ concentrations were at the high end of the previously reported values^32,33,36^. Similar to these other studies, the aragonite saturation state (Ω_Ar_) of ambient seawater at our study site was above saturation (i.e. 1.2 ± 0.02). The lowest pH recorded in the shallow coastal Ross Sea is >7.90 (in July^33^), although measurements from mid-winter are likely to be lower. Consequently, all three of our experimental pH simulations represent projected future scenarios, i.e. conditions outside those currently experienced in these coastal environments. All were also undersaturated with respect to aragonite (Table 1), as is projected to occur at times in several areas of the Southern Ocean (including the Ross Sea) in the next 8–10 years^11,12^.

Microalgal community health was evident from visual observations made through the clear Perspex chamber walls and from empirical PAM fluorometry measurements, confirming the suitability of our experimental system for growth and maintenance of these microbial communities. The maximum quantum yield (F_v_/F_m_) of Chl *a* fluorescence averaged 0.523 to 0.585 across the treatments, and was similar to spring-time values from other bottom-ice algae studies in this region (e.g.^15,37^). Chl *a* concentrations in our chambers were, at 30–55 mg Chl *a* m^−2^, well within the range of 4.4 to 173 mg Chl *a* m^−2^ measured in fast ice at nearby Cape Evans (McMurdo Sound, Ross Sea) in three separate years during spring^38^. C:N ratios were close to the classical Redfield (1963) ratio of 6.6^39^ (i.e., 10–12; Fig. 4d) and to measurements at the ice/water interface from two ice cores at nearby Cape Evans (8.6 and 8.3^40^).

Over the two week experiment, the response of the under ice algal community to our experimental treatments indicated an increase in productivity with reduced pH (Figs 2c and 3d), although the DO fluxes were variable and the trends were weak. pH increased (i.e. pCO_2_ concentrations declined) after 12 hours residence time in the chambers in all treatments relative to the inflow water (Fig. 3a–c; Table 1). In the 7.86 and 7.75 pH treatment chambers, outflow pH had increased to approximately ambient levels. In our most extreme treatment (pH 7.61), although outflow pH became elevated by 0.2 pH units relative to the inflow seawater (Table 1), average levels remained considerably lower (with higher pCO_2_ concentrations) than those of the ambient chambers (7.87 cf. 8.08, respectively; Table 1). Additionally, relative to the ambient treatment, the average carbonate saturation states in this lowest pH treatment were undersaturated for aragonite (Ω_Ar_ = 0.9 ± 0.10 vs 1.4 ± 0.05; Table 1) and considerably nearer to undersaturation for calcite (Ω_Ca_ = 1.7 + 0.16 vs 2.2 ± 0.08; Table 1).

In line with the relatively weak effect of pH treatment on primary productivity levels, there were no significant effects of reduced pH on C:N, POC, Chl *a*, Phaeo, Chl *a*:Phaeo or abundance of heterotrophic bacteria associated with the under ice microalgal community and sea ice platelet matrix (Fig. 4; Table 2). If microalgal CO_2_ fixation was the primary factor governing the observed pH change, under-ice POC concentrations would be expected to be significantly higher in the low pH treatments; yet this was not the case (Fig. 4f). There is a possibility that we have underestimated productivity, e.g. through enclosure of the sea ice/water within our large chambers we may have modified the circulation and the thickness of the sea ice diffusive boundary layer^41^, although care was taken to ensure the water in our chambers was well mixed at velocities close to ambient^34^. Estimates of CO_2_ fixation from our DO flux measurements (using a photosynthetic quotient of 1.03^32,42^) suggest a difference in C removal of ~200 mg C/m^2^ more in the pH 7.61 vs ambient pH treatment chambers, values in agreement with the magnitudes of difference noted in POC at the end of the experiment (Fig. 4f). This indicates that diffusion of CO_2_ from the chambers into the overlying ice may have occurred. While we could not quantify CO_2_ concentrations in the sea ice column above the chambers as part of this experiment, it is likely to have contained relatively high pH/low CO_2_^43,44^, creating a gradient with the underlying water that may have resulted in greater diffusion of CO_2_ out of the lower pH treatment chambers, and supporting the role of diffusion in altering the in-chamber pH.

Contrary to our findings, two laboratory studies investigating responses of the common sea ice diatom species *Nitzschia* sp. ICE-H and *Nitzschia lecointei* van Heurck 1909 to elevated pCO_2_ conditions have noted increases in bacterial growth^29^ and POC production^45^, associated with higher diatom growth rates. Similarly, bacterial abundances increased in another study in response to increased pCO_2_ in oceanic Ross Sea waters (not associated with sea ice)^46^. We did not see a positive relationship between bacterial abundance and elevated pCO_2_ in the bottom ice sampled from our experimental chambers, rather abundances were highly variable within and between treatments (Fig. 4e). The large variation in these sea ice community characteristics, both within our study and across other studies mentioned here, reflect the fact that sea ice and the associated microbial community is heterogeneous, across multiple scales (e.g.^15,37^). The contrasting results likely reflect the inclusion of natural sea ice habitat in our study, rather than isolated microalgae or water masses alone. More *in situ* investigations are required to understand how ocean acidification might affect the functioning of this system at different spatial scales and through the season, to reflect the fact that these environments are constantly changing during the sea ice growth and melt cycle.

We had anticipated shifts in microalgal community abundance and composition similar to those of studies of diatomaceous Southern Ocean phytoplankton (e.g. reviewed by^47^). However, across our pH treatments, the microalgal communities were comprised of similar numbers and types of taxa (Fig. 5). The lowest pH treatment contained the greatest and least prevalence of two common taxa, *Berkeleya adeliensis* and *Entomoneis kufferathii*, respectively, though there was no clear linear pattern in their abundances from high to low pH (Fig. 5). Similarly, the MDS illustrates a separation in ordination space of the community in the 7.61 pH treatment (Fig. 6), and the PERMANOVA indicates stronger differences in pairwise comparisons involving this lowest pH treatment than in comparisons involving any of the higher pH treatments. Studies of longer duration than the two weeks used here may be required to better understand the effects of ocean acidification on these communities and their composite species (c.f. 29), particularly considering the doubling times of sea ice algae (~5–10 d for McMurdo Sound sea ice microalgae^48^).

Although to our knowledge there are no comparable *in situ* investigations of ocean acidification on under-ice algae, context for our experimental results is provided by studies of coastal Antarctic phytoplankton^49,50^ and ice-algal productivity models^32^. Matson *et al*.^32^ predicted a resultant maximum oxygen production rate by sea ice algae of 5353 μmol m^−2^ h^−1^, which was about twice as high as the maximum daily oxygen production values that we estimated empirically using our DO logger data (~2500 μmol m^−2^ h^−1^; Fig. 2c), though their estimates were based on higher ice algal biomass (125 vs ~40 mg m^−2^; Fig. 4a) and thinner sea ice (1.75 m vs 2.0 m, respectively), and did not incorporate potential diffusion of pCO_2_ into the overlying sea ice layer. The estimates of the magnitudes of pH change due to photosynthesis by the algal community predicted by Matson *et al*.^32^ appear to be consistent with our results. However, the enhanced production noted in our chambers in response to low pH/high pCO_2_ conditions is counter to the findings of reduced photosynthesis and primary production of coastal phytoplankton with ocean acidification (at ~1140 μatm) reported recently elsewhere^49,50^.

Our experimental manipulations of pCO_2_ to this under sea ice environment had surprisingly little effect on the ice-associated microalgal community, suggesting it is relatively robust to low pH (at least over a two-week period). Our indirect measurements of CO_2_ uptake by the microalgae demonstrate the capacity of biological activity, in combination with non-biological sea-ice-seawater gas exchange, to modify effects of ocean acidification *in situ* (e.g.^32,33,43,44,51,52^). Nevertheless, when seawater pH is close to our most extreme levels tested (7.61), this combination of processes may not completely mitigate the effects of enhanced ocean CO_2_ concentrations; pH and carbonate saturation states remained low under this scenario. This is of concern for the structure and functioning of organisms that utilise the sea ice underside as a habitat (e.g.^53–55^). Importantly, these combined processes should be a key consideration in predictions of impacts of ocean acidification for these high latitude, ice covered regions.

## Experimental design and Methods

A 15 d long seawater manipulation experiment was conducted at Granite Harbour, Ross Sea (77° 00.963′S, 162° 52.607′E) from November 3^rd^ to 18^th^ 2014 (Day 0 to Day 14).

### Under-ice chambers

The underside of the sea ice, and the underlying water, were enclosed in transparent, flow-through incubation chambers^34^ (Fig. 1a,b). The open top of each chamber (70 cm diam. x 60 cm deep) was pushed up against the ice and the held firmly in place using air captured in the chamber’s buoyancy compartment (Fig. 1a; 34). The upward buoyant force of the of trapped air (~20 kg lift) created a mechanical seal between the chamber edge (seal) and the ice under-surface^34^. Each chamber enclosed a 0.36 m^2^ area of the sea ice-seawater interface and 144 L of the adjacent underlying seawater (Fig. 1b). Umbilical cables (Fig. 1a) supplied each of 16 chambers with seawater from header tanks located in an above ice laboratory (seawater supply rate = 200 mL min^−1^, chamber water residence time = ~12 h). Seawater was supplied to the chambers continually and exited the chambers via the exit port located on the chamber side^34^. To avoid stratification of seawater within the chambers at these low flow rates, the water was mixed at similar velocities to those naturally experienced at this site (as described in 34).

Chambers were deployed on Nov 2, and the flow of ambient seawater to each chamber initiated 45 minutes later. The following day (Day 0), the pH in the three treatment header tanks was gradually reduced over a 6 h period, with target pH levels obtained by 1600 h. Each header tank supplied four chambers, with the positions of individual chamber replicates randomly interspersed.

### Seawater pH manipulation

Four chambers were supplied with ambient pH seawater (pH 7.99), and four each with seawater at one of three reduced pH levels (7.86, 7.75, 7.61). The reduced pH concentrations were obtained via semi-continuous dosing of food grade CO_2_ via a submerged diffuser coil of thin-walled silicon tube in each header tank^34^. Header tanks contained a pH probe that allowed real-time monitoring of seawater pH, and daily water samples were measured spectrophotometrically to ensure target values were being maintained (methods below). Throughout this manuscript, pH is presented on the total hydrogen scale, at *in situ* temperature. The chambers assigned to a given treatment type were true independent replicates rather than pseudo-replicates^56–58^ because they were unable to influence each other when positioned on the undersurface of the ice, the four header tanks were identical in all regards (including being continually supplied by seawater and CO_2_ from single common sources), and because our assessments of chamber water conditions were made in each of the ~15 m long umbilical tubes which fed individual chambers. None of our measurements or observations suggested that there was anything substantially different about the header tanks (e.g. a contamination problem) other than the CO_2_ dose treatments we applied.

### Within-chamber measurements

Data loggers inside each chamber recorded DO (ZebraTech® D-Opto DO loggers), PAR (Odyssey® light loggers) and temperature (Seabird® SBE 56) at ≤10 min intervals throughout the experiment. Temperature and DO-loggers were also deployed in each of the four header tanks. Dissolved oxygen concentrations in all chambers at our site were high (>10 mg L^−1^) but never exceeded 90% saturation.

### Characterising chamber inflow and outflow water

Daily measurements of water being delivered to the chambers (inflow), and water inside the chambers (outflow) determined pH, salinity and concentrations of DO and inorganic nutrients (DIN, NH_4_^+^, NO_3_^−^, DRP; Si(OH)_4_). Samples were collected at 0900 h on Day 0, and thereafter at ~1400 h each day, to avoid any potential confounding of results with temporal differences in light and biological activity. At each time point, two 60 ml samples were collected from the inflow water and two 60 ml samples from the outflow water (see^34^). For each water type, one 60 ml sample was used to measure concentrations of DO and nutrients and the other for determination of pH and salinity. Although samples were collected, filtered and frozen every day, within-chamber nutrients were not analysed on all dates. However, NH_4_^+^_,_ NO_3_^−^, and DRP concentrations in ambient inflow seawater samples were assessed every day.

An automated spectrophotometric system and thymol blue indicator dye was used to measure pH_T_ (detailed in^59^). Mean pH for each treatment across the experiment is shown in Table 1. Salinity was determined using a HACH HQ40d, with HACCDC401-01 conductivity probe.

DO concentrations were determined from each sample using an optical DO probe. Samples were immediately filtered (GF/C Millipore), and the water frozen and stored in the dark until later analysis to determine dissolved inorganic nutrient concentrations using standard methods for seawater (Astoria-Pacific 300 series segmented flow auto-analyser; detection limits of 1 mg m^−3^ for N and P).

Additional outflow water samples were collected on Days 0, 1, 7 and 14 and preserved with HgCl_2_ for analysis of alkalinity (A_T_). A_T_ was determined using a closed cell potentiometric titration method^60^, the accuracy of which is estimated to be 1.5 μmol kg^−1^, based on analyses of Certified Reference Material supplied by Andrew Dickson. pCO_2_ concentrations were calculated from measured A_T_ and pH at *in situ* water temperature and salinity, using refitted^61^ equilibrium constants^62^.

### End of experiment sampling

At the end of the experiment divers carefully removed the chambers and collected under-ice microalgal material and associated fauna across the central diameter of each chamber (10 wide x 70 cm long scrape). This method sampled the most active biological layer beneath the impenetrable hard ice, including all biota present in 1–2 cm of “soft” bottom ice and in the platelet ice layer that extended a few cm beneath. The 10 cm width mouth of a 2200 ml plastic sampling container was held firm against the hard underside of the ice and carefully moved across the diameter of the chamber footprint (70 cm) to collect all ice crystals and associated microalgae. The sampling container had mesh-covered holes at the bottom (22 μm mesh), allowing seawater to flow through while collecting and retaining all ice crystals and associated microalgae. The containers were capped immediately, brought to the surface, and held in the dark in a water bath with circulating ambient seawater to maintain *in situ* ambient temperatures.

After a dark adaption period of 30 to 60 minutes, the photosynthetic activity of the sea-ice microalgal material from each chamber was assessed using a monitoring Pulse-amplitude modulated (PAM) chlorophyll fluorometer (Walz, Moni-PAM). At the time of processing, sample temperatures ranged from −0.90 to −1.70 °C (average −1.58 °C), and salinities from 34.2 to 32.8‰ (average 34.0‰). Four measurements of the maximum quantum yield of Chlorophyll *a* photosystem II (Φ_PSII_), F_v_/F_m_ were made in each container under steady state illumination at low light levels, using a weak (<1 μmol photons m^−1^ s^−1^) blue LED measuring and actinic light, to reflect relative *in situ* algal photosynthetic competence. Multiple measurements were taken from each container to better reflect the large volume of the sample (~2200 ml), and subsequently combined to give an average F_V_/F_M_ that was then compared among experimental treatments.

Once PAM measurements were complete, samples were homogenised, subsampled and preserved as follows: algal community composition (60 ml, non-acidified Lugols iodine, stored in darkness); abundance of heterotrophic bacteria (3 × 1 ml, snap frozen in liquid N_2_); Chl *a* and Phaeo, and particulate N and C (150 ml each, GF/F Millipore, frozen −20 °C, stored in darkness); particulate organic carbon (POC; 150 ml, precombusted GF/F, frozen −20 °C). Quantification of algal community composition (species, abundance) was assessed using optical microscopy. A 2 ml subsample was settled for a minimum of 4 h and examined in Utermohl chambers on a Leitz inverted microscope. Cell densities of heterotrophic bacteria (number mL^−1^, average of triplicate samples) were determined using Flow Cytometry after first pre-filtering (20 μm). Chl *a* was extracted from the filters using 90% acetone, and the extract measured using spectrofluorometry on a Varian Cary spectrofluorometer. An acidification step was used to correct for phaeophytin interference, and to thus determine Phaeo concentration^63,64^. PN, PC and POC were analysed using high temperature combustion (furnace at ca. 1000 °C) in the presence of a catalyst to convert Carbon to CO_2_ and Nitrogen to N_2_, following standard procedures^65^. Analyses were performed using an Elementar Vario EL 111. For PN and PC, the filters were wrapped in tin foil prior to combustion, and calibration for each element used high purity acetanilide. For POC, sulphuric acid was first added to the filter to remove inorganic substances. Separation of the gases occurred using a chromatographic column and were determined in succession with a Thermal Conductivity Detector.

### Statistical analysis

Although four nominally categorical pH treatments were maintained for 15 d, measured inflow pH was able to be used as a continuous independent predictor variable in some analyses.

Several variables measured daily were analysed as responses, for example, DO fluxes were indicative of net photosynthetic oxygen production by the under ice community^66^, ΔpH (outflow pH minus inflow pH) was indicative of CO_2_ loss, incorporating both biological (photosynthesis) and non-biological losses (diffusion into the ice above). Fluxes of DO were calculated as Concentration_outflow_ minus Concentration_inflow_, multiplied by seawater supply rate^67^ and standardised by the area of under ice algal habitat enclosed by each chamber (units of μmol O_2_ m^−2^ h^−1^).

DO fluxes were also able to be calculated from the DO logger data collected synchronously at 10-minute intervals in all chambers and header tanks. Fluxes were calculated at each time interval from the differences in DO concentration between a chamber’s logger (representing outflow) and the average of the four header tank loggers (representing inflow). Although the DO loggers were not reliable in all cases, datalogger data from two to three replicates per treatment type were able to be utilised for plotting and analysis.

As light and temperature can influence photosynthetic rates, logged data were used as explanatory variables. We averaged the quantity of light recorded by all above and under-ice PAR sensors between 00:00 to 08:00 h. The ratio of under-ice to above-ice PAR was used to capture the changes in incident light in combination with factors that had the potential to affect under-ice PAR sensor readings (e.g. changes in algal biomass, or detritus settling on the up-facing sensors). Seawater temperature data between 00:00 to 08:00 were also averaged. The 00:00 to 08:00 averages were used as predictors of DO flux and ΔpH responses assessed at 14:00 h because the high frequency DO logger data showed photosynthetic peaks occurred six hours later than peaks in incident sunlight intensity (six hours is exactly one-half of the water residence time of the chambers).

To statistically evaluate DO flux and ΔpH responses, we used daily pH inflow values and days from the start of the experiment as continuous independent variables in simple generalised linear models (Proc GLM, SAS 9.3). The interaction between pH inflow and day of experiment was calculated by standardising and centring each variable and multiplying them together. We progressed to multiple regression analysis to simultaneously examine the influence of multiple factors (Proc REG, SAS 9.3). All explanatory variables (inflow pH, day, under- and above-ice PAR, under:above-ice PAR ratio, *in situ* seawater temperature, ambient seawater concentrations of P, NH_4_^+^, NO_3_^−^, DIN, and N:P ratio) were standardised to run between 0 and 1. Variables were eliminated from full models using a backward selection procedure (selection criterion α = 0.15; final model significance level α = 0.05). Collinearity diagnostics and variance inflation factors were examined, homogeneity of variance was evaluated by plotting residual vs. predicted values, and normality was assessed via normal probability plots and Shapiro-Wilk tests on residuals to ensure that the final retained models met the assumptions of the tests, which they did.

Samples collected at the end of the experiment documented the cumulative effect of the pH treatments that were maintained for 15 d. Univariate data (Chl *a*, Phaeo, Chl *a*:Phaeo, C:N, POC, heterotrophic bacterial abundance, F_v_/F_m_) were analysed using permutational distance-based multivariate analysis of variance (PERMANOVA; PRIMER 7^35^), with pairwise comparisons to identify significant differences in between pH treatments. Microalgal community composition data were investigated using PERMANOVA based on Bray‐Curtis dissimilarities of untransformed and square root transformed data, followed by pairwise comparisons. Species contributions to the dissimilarity/similarity among treatments were identified using SIMPER, and illustrated using non-metric multidimensional scaling (MDS; PRIMER 7^35^).

## Supplementary information

Supplementary Table 1

## Data Availability

The data sets generated during this study are available from the corresponding author upon reasonable request.
