# Supplementary material for: In situ response of Antarctic under-ice primary producers to experimentally altered pH
Source: Sci Rep. 2019 Apr 15;9:6069. doi: 10.1038/s41598-019-42329-0 (PMC6465331; doi:10.1038/s41598-019-42329-0)
Supplement: Supplementary file 1 — Supplementary Table 1 [file 41598_2019_42329_MOESM1_ESM.docx]

**Supplementary Information**

***In situ* response of Antarctic under-ice primary producers to experimentally altered pH**

Vonda J. Cummings, Neill G. Bar^r^, Rod G. Budd, Peter M. Marriott, Karl A. Safi, & Andrew M. Lohrer

**Supplementary Table 1**. The microalgal taxa contributing to 90% of the variability in assemblage composition in the ice scrapes (from SIMPER analysis of untransformed data). For each treatment, the taxa ranking is shown, with the percentage contribution of each taxa to assemblage variability given in parentheses.

| **Taxa group** | **pH 7.99** | **pH 7.86** | **pH 7.75** | **pH 7.61** |
| --- | --- | --- | --- | --- |
| % within treatment similarity | 72.07% | 69.61% | 81.62% | 82.63% |
| *Berkeleya adeliensis* | 1 (37.35) | 1 (43.91) | 1 (33.36) | 1 (55.15) |
| *Entomoneis kufferathii* | 2 (30.73) | 2 (29.71) | 2 (32.09) | 2 (22.35) |
| *Nitzschia* spp. | 3 (6.51) | 4 (4.83) | 4 (5.72) | 3 (3.96) |
| *Navicula* spp. | 4 (6.45) | 3 (8.74) | 3 (12.66) | 5 (3.16) |
| *Nitzschia stellata* | 5 (5.66) | 5 (3.84) | 5 (3.27) | 4 (3.54) |
| *Haslea* sp. | 6 (4.32) |  | 6 (3.05) |  |
| *Cylindrotheca closterium* |  |  |  | 6 (2.94) |
